# 2-[1-(3-{2-[(2-Hy­droxy­benzyl­idene)amino]­phen­oxy}prop­yl)-1*H*-1,3-benzodiazol-2-yl]phenol

**DOI:** 10.1107/S1600536811005319

**Published:** 2011-02-19

**Authors:** Hassan Keypour, Sareh Tamizi, Saeed Dehghanpour, Reza Azadbakht, Mehdi Khalaj

**Affiliations:** a Faculty of Chemistry, Bu-Ali Sina University, Hamedan 65174, Iran; b Department of Chemistry, Alzahra University, Vanak, Tehran, Iran; c Department of Chemistry, Payam Noor University, Hamedan, Iran; d Department of Chemistry, Islamic Azad University, Buinzahra Branch, Buinzahra, Qazvin, Iran

## Abstract

In the title compound, C_29_H_25_N_3_O_3_, the imine double bond has an *E* configuration. The dihedral angle between the hy­droxy­phenyl and benzene rings in the imine moiety is 26.95 (9)°, and the dihedral angle between the hy­droxy­phenyl and benzimidazole rings in the other moiety is 14.83 (9)°. These angles are probably limited to small values as a consequence of two strong intra­molecular O—H⋯N hydrogen bonds formed between the hy­droxy groups and the imine and imidazole N atoms. The aliphatic chain linking the two ring systems has a *gauche* conformation, as reflected in C—C—C—O torsion angle of 70.9 (2)°.

## Related literature

For related structures, see: Keypour *et al.* (2009 ▶). For background information on diimine complexes, see: Mahmoudi *et al.* (2009 ▶). 
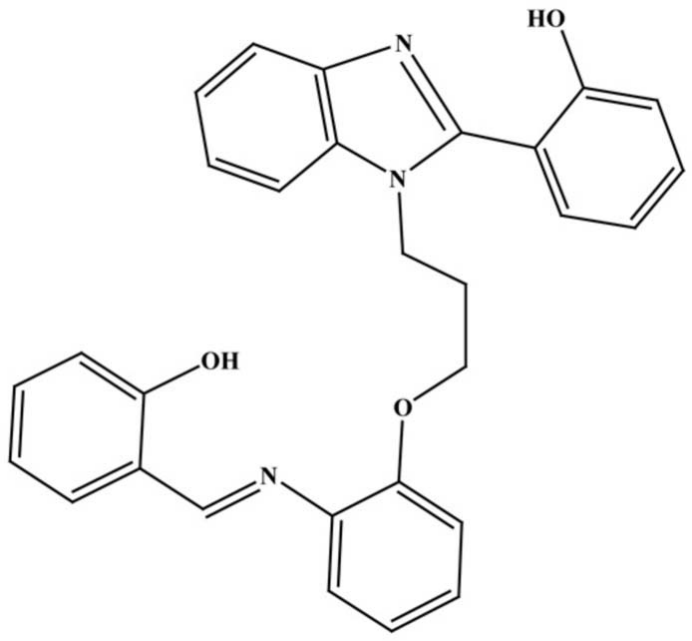


## Experimental

### 

#### Crystal data

C_29_H_25_N_3_O_3_
*M* *_r_* = 463.52
Monoclinic, 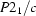

*a* = 9.1097 (6) Å
*b* = 18.1946 (11) Å
*c* = 13.7769 (5) Å
β = 93.405 (4)°
*V* = 2279.5 (2) Å^3^
*Z* = 4
Mo *K*α radiation
μ = 0.09 mm^−1^
*T* = 150 K
0.25 × 0.12 × 0.10 mm

#### Data collection

Nonius KappaCCD diffractometer
Absorption correction: multi-scan (*SORTAV*; Blessing, 1995 ▶) *T*_min_ = 0.866, *T*_max_ = 0.993
16359 measured reflections
5135 independent reflections
3083 reflections with *I* > 2σ(*I*)
*R*_int_ = 0.054

#### Refinement

*R*[*F* ^2^ > 2σ(*F* ^2^)] = 0.055
*wR*(*F* ^2^) = 0.137
*S* = 1.06
5135 reflections
318 parameters
1 restraint
H-atom parameters constrained
Δρ_max_ = 0.23 e Å^−3^
Δρ_min_ = −0.20 e Å^−3^

### 

Data collection: *COLLECT* (Nonius, 2002 ▶); cell refinement: *DENZO-SMN* (Otwinowski & Minor, 1997 ▶); data reduction: *DENZO-SMN*; program(s) used to solve structure: *SIR92* (Altomare *et al.*, 1994 ▶); program(s) used to refine structure: *SHELXTL* (Sheldrick, 2008 ▶); molecular graphics: *PLATON* (Spek, 2009 ▶); software used to prepare material for publication: *SHELXTL*.

## Supplementary Material

Crystal structure: contains datablocks I, global. DOI: 10.1107/S1600536811005319/bh2338sup1.cif

Structure factors: contains datablocks I. DOI: 10.1107/S1600536811005319/bh2338Isup2.hkl

Additional supplementary materials:  crystallographic information; 3D view; checkCIF report

## Figures and Tables

**Table 1:** Hydrogen-bond geometry (Å, °)

*D*—H⋯*A*	*D*—H	H⋯*A*	*D*⋯*A*	*D*—H⋯*A*
O1—H1*O*⋯N2	0.84	1.81	2.564 (2)	148
O3—H2*O*⋯N3	0.84	1.80	2.548 (2)	148

**Fig. 1.**
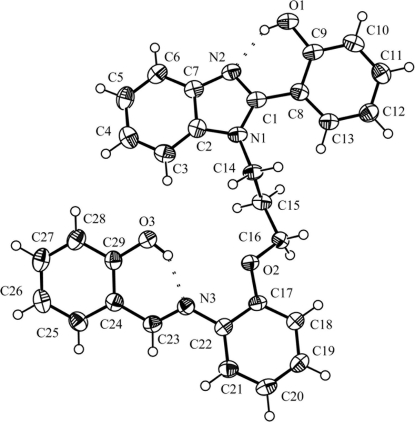
A view of the structure of the title complex, with displacement ellipsoids drawn at the 50% probability level.

## supplementary crystallographic information

### Comment

In our ongoing studies on the synthesis, structural and spectroscopic characterization of the products derived from *N*^1^-(3-(2-aminophenoxy)propyl)-benzene-1,2-diamine with aldehydes (Keypour *et al.*, 2009; Mahmoudi *et al.*, 2009) we report herein the crystal structure of the title compound, prepared by the reaction of *N*^1^-(3-(2-aminophenoxy)propyl)-benzene-1,2-diamine with salicyl aldehyde.

The molecular structure of the title compound is shown in Fig. 1. The molecule adopts the *E* configuration with respect to the imine C═N bond. Two hydroxyl groups are located close to N atoms, and form strong intramolecular hydrogen bonds (Table 1).

### Experimental

*N*^1^-(3-(2-aminophenoxy)propyl)benzene-1,2-diamine (0.064 g, 0.25 mmol) in methanol (20 ml) was added dropwise with stirring to a solution of salicylaldehyde (0.061 g, 0.5 mmol) in methanol (30 ml). The mixture was refluxed for 12 h. Then, the solution volume was reduced to 10 ml by evaporation, and a precipitate was formed. This was filtered off, washed with ether, and dried *in vacuo*. Vapour diffusion of diethyl ether into a methanolic solution of the product afforded yellow crystals in 60% yield.

### Refinement

All C-bonded H atoms positions were calculated and refined with a riding model and *U*_iso_(H) parameters set to 1.2 times *U*_eq_(carrier C atom). Hydroxyl H atoms also ride on their O atoms, with O—H bond lengths fixed to 0.84 Å and *U*_iso_(H) = 1.5 *U*_eq_(carrier O atom).

### Crystal data

**Table:**

C_29_H_25_N_3_O_3_	*F*(000) = 976
*M**_r_* = 463.52	*D*_x_ = 1.351 Mg m^−^^3^
Monoclinic, *P*2_1_/*c*	Mo *K*α radiation, λ = 0.71073 Å
Hall symbol: -P 2ybc	Cell parameters from 16359 reflections
*a* = 9.1097 (6) Å	θ = 2.7–27.5°
*b* = 18.1946 (11) Å	µ = 0.09 mm^−^^1^
*c* = 13.7769 (5) Å	*T* = 150 K
β = 93.405 (4)°	Needle, yellow
*V* = 2279.5 (2) Å^3^	0.25 × 0.12 × 0.10 mm
*Z* = 4	

### Data collection

**Table:**

Nonius KappaCCD diffractometer	5135 independent reflections
Radiation source: fine-focus sealed tube	3083 reflections with *I* > 2σ(*I*)
graphite	*R*_int_ = 0.054
Detector resolution: 9 pixels mm^-1^	θ_max_ = 27.5°, θ_min_ = 2.7°
φ scans and ω scans with κ offsets	*h* = −11→11
Absorption correction: multi-scan (*SORTAV*; Blessing, 1995)	*k* = −22→23
*T*_min_ = 0.866, *T*_max_ = 0.993	*l* = −17→17
16359 measured reflections	

### Refinement

**Table:**

Refinement on *F*^2^	Primary atom site location: structure-invariant direct methods
Least-squares matrix: full	Secondary atom site location: difference Fourier map
*R*[*F*^2^ > 2σ(*F*^2^)] = 0.055	Hydrogen site location: inferred from neighbouring sites
*wR*(*F*^2^) = 0.137	H-atom parameters constrained
*S* = 1.06	*w* = 1/[σ^2^(*F*_o_^2^) + (0.0536*P*)^2^ + 0.4485*P*] where *P* = (*F*_o_^2^ + 2*F*_c_^2^)/3
5135 reflections	(Δ/σ)_max_ = 0.001
318 parameters	Δρ_max_ = 0.23 e Å^−^^3^
1 restraint	Δρ_min_ = −0.20 e Å^−^^3^
0 constraints	

### Fractional atomic coordinates and isotropic or equivalent isotropic displacement parameters (Å^2^)

**Table:**

	*x*	*y*	*z*	*U*_iso_*/*U*_eq_	
O1	0.98262 (18)	0.22668 (8)	0.88875 (9)	0.0465 (4)	
H1O	0.9212	0.1922	0.8864	0.070*	
O2	0.80830 (16)	0.02045 (7)	0.35975 (9)	0.0369 (4)	
O3	0.69646 (17)	−0.13681 (8)	0.49252 (9)	0.0418 (4)	
H2O	0.7212	−0.1156	0.4418	0.063*	
N1	0.82575 (18)	0.08636 (9)	0.66217 (11)	0.0352 (4)	
N2	0.80517 (19)	0.13040 (9)	0.81279 (11)	0.0378 (4)	
N3	0.70199 (18)	−0.10938 (9)	0.31140 (11)	0.0323 (4)	
C1	0.8674 (2)	0.13987 (11)	0.72906 (13)	0.0348 (5)	
C2	0.7258 (2)	0.04118 (11)	0.70695 (14)	0.0355 (5)	
C3	0.6454 (2)	−0.01894 (12)	0.67220 (16)	0.0424 (5)	
H3A	0.6527	−0.0369	0.6079	0.051*	
C4	0.5538 (2)	−0.05139 (12)	0.73642 (17)	0.0461 (6)	
H4A	0.4961	−0.0926	0.7155	0.055*	
C5	0.5439 (2)	−0.02520 (12)	0.83139 (17)	0.0462 (6)	
H5A	0.4805	−0.0492	0.8736	0.055*	
C6	0.6241 (2)	0.03446 (12)	0.86451 (16)	0.0428 (5)	
H6A	0.6173	0.0520	0.9290	0.051*	
C7	0.7156 (2)	0.06849 (11)	0.80071 (14)	0.0371 (5)	
C8	0.9683 (2)	0.20146 (11)	0.71522 (14)	0.0342 (5)	
C9	1.0249 (2)	0.24102 (12)	0.79778 (14)	0.0358 (5)	
C10	1.1272 (2)	0.29677 (12)	0.78825 (15)	0.0406 (5)	
H10A	1.1682	0.3211	0.8445	0.049*	
C11	1.1694 (2)	0.31703 (12)	0.69784 (16)	0.0433 (5)	
H11A	1.2402	0.3549	0.6920	0.052*	
C12	1.1092 (2)	0.28247 (12)	0.61553 (15)	0.0411 (5)	
H12A	1.1353	0.2979	0.5530	0.049*	
C13	1.0116 (2)	0.22574 (11)	0.62434 (14)	0.0375 (5)	
H13A	0.9720	0.2021	0.5671	0.045*	
C14	0.8752 (2)	0.07122 (11)	0.56509 (13)	0.0354 (5)	
H14A	0.8771	0.0174	0.5545	0.042*	
H14B	0.9765	0.0900	0.5607	0.042*	
C15	0.7747 (2)	0.10687 (12)	0.48571 (13)	0.0372 (5)	
H15A	0.6749	0.0856	0.4877	0.045*	
H15B	0.7675	0.1602	0.4991	0.045*	
C16	0.8287 (2)	0.09626 (11)	0.38527 (14)	0.0375 (5)	
H16A	0.7725	0.1280	0.3380	0.045*	
H16B	0.9341	0.1095	0.3847	0.045*	
C17	0.8190 (2)	0.00152 (11)	0.26392 (13)	0.0319 (5)	
C18	0.8766 (2)	0.04669 (12)	0.19473 (14)	0.0382 (5)	
H18A	0.9154	0.0936	0.2127	0.046*	
C19	0.8774 (2)	0.02298 (12)	0.09868 (15)	0.0420 (5)	
H19A	0.9156	0.0542	0.0510	0.050*	
C20	0.8233 (2)	−0.04524 (12)	0.07220 (14)	0.0408 (5)	
H20A	0.8249	−0.0611	0.0066	0.049*	
C21	0.7667 (2)	−0.09051 (12)	0.14125 (14)	0.0367 (5)	
H21A	0.7311	−0.1379	0.1230	0.044*	
C22	0.7614 (2)	−0.06751 (11)	0.23715 (13)	0.0320 (5)	
C23	0.5987 (2)	−0.15625 (11)	0.29380 (14)	0.0344 (5)	
H23A	0.5604	−0.1634	0.2288	0.041*	
C24	0.5396 (2)	−0.19841 (11)	0.37172 (14)	0.0339 (5)	
C25	0.4292 (2)	−0.25061 (12)	0.35230 (16)	0.0403 (5)	
H25A	0.3930	−0.2584	0.2870	0.048*	
C26	0.3717 (2)	−0.29106 (12)	0.42569 (17)	0.0458 (6)	
H26A	0.2965	−0.3263	0.4114	0.055*	
C27	0.4258 (2)	−0.27940 (12)	0.52143 (16)	0.0444 (6)	
H27A	0.3875	−0.3073	0.5725	0.053*	
C28	0.5338 (2)	−0.22813 (12)	0.54275 (15)	0.0407 (5)	
H28A	0.5687	−0.2206	0.6083	0.049*	
C29	0.5923 (2)	−0.18717 (11)	0.46906 (14)	0.0340 (5)	

### Atomic displacement parameters (Å^2^)

**Table:**

	*U*^11^	*U*^22^	*U*^33^	*U*^12^	*U*^13^	*U*^23^
O1	0.0556 (11)	0.0529 (11)	0.0310 (8)	−0.0016 (8)	0.0015 (7)	−0.0072 (7)
O2	0.0491 (9)	0.0346 (8)	0.0271 (7)	−0.0053 (7)	0.0029 (6)	−0.0018 (6)
O3	0.0502 (10)	0.0431 (9)	0.0320 (8)	−0.0091 (7)	0.0014 (6)	0.0014 (6)
N1	0.0373 (10)	0.0394 (10)	0.0289 (9)	0.0047 (8)	0.0017 (7)	−0.0045 (7)
N2	0.0435 (11)	0.0402 (11)	0.0298 (9)	0.0039 (9)	0.0032 (7)	−0.0015 (7)
N3	0.0351 (10)	0.0305 (10)	0.0314 (9)	0.0023 (8)	0.0033 (7)	0.0000 (7)
C1	0.0339 (12)	0.0397 (13)	0.0307 (11)	0.0079 (9)	−0.0001 (8)	−0.0026 (9)
C2	0.0312 (12)	0.0353 (12)	0.0401 (12)	0.0057 (9)	0.0023 (9)	0.0017 (9)
C3	0.0427 (13)	0.0416 (14)	0.0423 (13)	0.0072 (11)	−0.0021 (10)	−0.0059 (10)
C4	0.0393 (14)	0.0382 (13)	0.0604 (15)	0.0013 (11)	−0.0010 (11)	−0.0007 (11)
C5	0.0420 (14)	0.0399 (14)	0.0577 (15)	0.0057 (11)	0.0120 (11)	0.0111 (11)
C6	0.0461 (14)	0.0430 (14)	0.0398 (12)	0.0082 (11)	0.0076 (10)	0.0045 (10)
C7	0.0366 (12)	0.0392 (13)	0.0354 (12)	0.0060 (10)	0.0017 (9)	−0.0001 (9)
C8	0.0298 (11)	0.0366 (12)	0.0358 (11)	0.0035 (9)	−0.0005 (8)	−0.0022 (9)
C9	0.0358 (12)	0.0432 (13)	0.0283 (11)	0.0114 (10)	0.0000 (8)	−0.0022 (9)
C10	0.0368 (13)	0.0430 (13)	0.0410 (13)	0.0051 (11)	−0.0046 (9)	−0.0104 (10)
C11	0.0356 (13)	0.0408 (13)	0.0533 (14)	0.0008 (10)	0.0021 (10)	−0.0037 (10)
C12	0.0413 (13)	0.0444 (14)	0.0380 (12)	0.0029 (11)	0.0062 (9)	−0.0009 (10)
C13	0.0387 (13)	0.0415 (13)	0.0321 (11)	0.0024 (10)	0.0001 (9)	−0.0048 (9)
C14	0.0390 (12)	0.0399 (12)	0.0275 (11)	0.0067 (10)	0.0041 (8)	−0.0067 (9)
C15	0.0392 (12)	0.0392 (13)	0.0327 (11)	0.0039 (10)	−0.0007 (9)	−0.0045 (9)
C16	0.0437 (13)	0.0349 (12)	0.0335 (11)	−0.0050 (10)	−0.0003 (9)	−0.0007 (9)
C17	0.0323 (11)	0.0390 (12)	0.0244 (10)	0.0016 (9)	0.0011 (8)	−0.0013 (8)
C18	0.0417 (13)	0.0408 (13)	0.0325 (12)	−0.0043 (10)	0.0051 (9)	0.0012 (9)
C19	0.0448 (14)	0.0487 (14)	0.0334 (12)	−0.0011 (11)	0.0102 (9)	0.0040 (10)
C20	0.0458 (14)	0.0488 (14)	0.0286 (11)	0.0046 (11)	0.0080 (9)	−0.0028 (9)
C21	0.0398 (13)	0.0366 (13)	0.0336 (11)	0.0047 (10)	0.0024 (9)	−0.0041 (9)
C22	0.0308 (11)	0.0342 (12)	0.0310 (11)	0.0050 (9)	0.0027 (8)	0.0025 (8)
C23	0.0376 (12)	0.0333 (12)	0.0322 (11)	0.0058 (10)	0.0019 (9)	−0.0013 (9)
C24	0.0346 (12)	0.0297 (11)	0.0377 (12)	0.0050 (9)	0.0040 (9)	−0.0009 (9)
C25	0.0384 (13)	0.0353 (12)	0.0471 (13)	0.0004 (10)	0.0025 (10)	−0.0056 (10)
C26	0.0382 (14)	0.0345 (13)	0.0649 (16)	−0.0034 (10)	0.0058 (11)	0.0011 (11)
C27	0.0413 (14)	0.0381 (13)	0.0549 (14)	0.0044 (11)	0.0116 (10)	0.0133 (10)
C28	0.0406 (13)	0.0407 (13)	0.0414 (12)	0.0051 (11)	0.0061 (9)	0.0085 (10)
C29	0.0330 (12)	0.0295 (12)	0.0397 (12)	0.0026 (9)	0.0043 (9)	0.0005 (9)

### Geometric parameters (Å, °)

**Table:**

O1—C9	1.358 (2)	C12—H12A	0.9500
O1—H1O	0.8400	C13—H13A	0.9500
O2—C17	1.373 (2)	C14—C15	1.528 (3)
O2—C16	1.433 (2)	C14—H14A	0.9900
O3—C29	1.344 (2)	C14—H14B	0.9900
O3—H2O	0.8400	C15—C16	1.508 (3)
N1—C1	1.378 (2)	C15—H15A	0.9900
N1—C2	1.397 (3)	C15—H15B	0.9900
N1—C14	1.462 (2)	C16—H16A	0.9900
N2—C1	1.326 (2)	C16—H16B	0.9900
N2—C7	1.395 (3)	C17—C18	1.385 (3)
N3—C23	1.282 (2)	C17—C22	1.402 (3)
N3—C22	1.409 (2)	C18—C19	1.392 (3)
C1—C8	1.469 (3)	C18—H18A	0.9500
C2—C3	1.386 (3)	C19—C20	1.377 (3)
C2—C7	1.392 (3)	C19—H19A	0.9500
C3—C4	1.384 (3)	C20—C21	1.381 (3)
C3—H3A	0.9500	C20—H20A	0.9500
C4—C5	1.400 (3)	C21—C22	1.390 (3)
C4—H4A	0.9500	C21—H21A	0.9500
C5—C6	1.371 (3)	C23—C24	1.449 (3)
C5—H5A	0.9500	C23—H23A	0.9500
C6—C7	1.392 (3)	C24—C25	1.397 (3)
C6—H6A	0.9500	C24—C29	1.412 (3)
C8—C13	1.406 (3)	C25—C26	1.379 (3)
C8—C9	1.417 (3)	C25—H25A	0.9500
C9—C10	1.388 (3)	C26—C27	1.397 (3)
C10—C11	1.376 (3)	C26—H26A	0.9500
C10—H10A	0.9500	C27—C28	1.374 (3)
C11—C12	1.381 (3)	C27—H27A	0.9500
C11—H11A	0.9500	C28—C29	1.391 (3)
C12—C13	1.372 (3)	C28—H28A	0.9500
			
C9—O1—H1O	109.5	H14A—C14—H14B	107.9
C17—O2—C16	117.50 (14)	C16—C15—C14	112.85 (17)
C29—O3—H2O	109.5	C16—C15—H15A	109.0
C1—N1—C2	106.32 (16)	C14—C15—H15A	109.0
C1—N1—C14	131.09 (17)	C16—C15—H15B	109.0
C2—N1—C14	122.48 (16)	C14—C15—H15B	109.0
C1—N2—C7	106.17 (16)	H15A—C15—H15B	107.8
C23—N3—C22	122.11 (16)	O2—C16—C15	107.68 (16)
N2—C1—N1	112.02 (18)	O2—C16—H16A	110.2
N2—C1—C8	121.00 (17)	C15—C16—H16A	110.2
N1—C1—C8	126.98 (18)	O2—C16—H16B	110.2
C3—C2—C7	122.6 (2)	C15—C16—H16B	110.2
C3—C2—N1	131.05 (19)	H16A—C16—H16B	108.5
C7—C2—N1	106.32 (18)	O2—C17—C18	124.33 (18)
C4—C3—C2	116.2 (2)	O2—C17—C22	115.51 (17)
C4—C3—H3A	121.9	C18—C17—C22	120.12 (17)
C2—C3—H3A	121.9	C17—C18—C19	119.6 (2)
C3—C4—C5	121.8 (2)	C17—C18—H18A	120.2
C3—C4—H4A	119.1	C19—C18—H18A	120.2
C5—C4—H4A	119.1	C20—C19—C18	120.6 (2)
C6—C5—C4	121.2 (2)	C20—C19—H19A	119.7
C6—C5—H5A	119.4	C18—C19—H19A	119.7
C4—C5—H5A	119.4	C19—C20—C21	119.83 (19)
C5—C6—C7	118.0 (2)	C19—C20—H20A	120.1
C5—C6—H6A	121.0	C21—C20—H20A	120.1
C7—C6—H6A	121.0	C20—C21—C22	120.7 (2)
C6—C7—C2	120.2 (2)	C20—C21—H21A	119.6
C6—C7—N2	130.68 (19)	C22—C21—H21A	119.6
C2—C7—N2	109.13 (17)	C21—C22—C17	119.08 (18)
C13—C8—C9	116.56 (19)	C21—C22—N3	124.35 (18)
C13—C8—C1	124.48 (18)	C17—C22—N3	116.57 (16)
C9—C8—C1	118.95 (18)	N3—C23—C24	120.85 (18)
O1—C9—C10	117.15 (18)	N3—C23—H23A	119.6
O1—C9—C8	122.22 (19)	C24—C23—H23A	119.6
C10—C9—C8	120.63 (18)	C25—C24—C29	118.71 (18)
C11—C10—C9	120.45 (19)	C25—C24—C23	120.85 (18)
C11—C10—H10A	119.8	C29—C24—C23	120.43 (18)
C9—C10—H10A	119.8	C26—C25—C24	121.5 (2)
C10—C11—C12	120.2 (2)	C26—C25—H25A	119.2
C10—C11—H11A	119.9	C24—C25—H25A	119.2
C12—C11—H11A	119.9	C25—C26—C27	118.9 (2)
C13—C12—C11	119.8 (2)	C25—C26—H26A	120.6
C13—C12—H12A	120.1	C27—C26—H26A	120.6
C11—C12—H12A	120.1	C28—C27—C26	120.9 (2)
C12—C13—C8	122.18 (18)	C28—C27—H27A	119.6
C12—C13—H13A	118.9	C26—C27—H27A	119.6
C8—C13—H13A	118.9	C27—C28—C29	120.5 (2)
N1—C14—C15	111.78 (16)	C27—C28—H28A	119.7
N1—C14—H14A	109.3	C29—C28—H28A	119.7
C15—C14—H14A	109.3	O3—C29—C28	119.03 (18)
N1—C14—H14B	109.3	O3—C29—C24	121.48 (18)
C15—C14—H14B	109.3	C28—C29—C24	119.48 (19)

### Hydrogen-bond geometry (Å, °)

**Table:**

*D*—H···*A*	*D*—H	H···*A*	*D*···*A*	*D*—H···*A*
O1—H1O···N2	0.84	1.81	2.564 (2)	148
O3—H2O···N3	0.84	1.80	2.548 (2)	148

## References

[bb1] Altomare, A., Cascarano, G., Giacovazzo, C., Guagliardi, A., Burla, M. C., Polidori, G. & Camalli, M. (1994). *J. Appl. Cryst.* **27**, 435.

[bb2] Blessing, R. H. (1995). *Acta Cryst.* A**51**, 33–38. 10.1107/s0108767394005726 7702794

[bb3] Keypour, H., Azadbakht, R., Salehzadeh, S., Rudbari, H. A. & Adams, H. (2009). *Tetrahedron Lett.* **50**, 169–171.

[bb4] Mahmoudi, A., Dehghanpour, S., Khalaj, M. & Pakravan, S. (2009). *Acta Cryst.* E**65**, m889. 10.1107/S1600536809025641 PMC2977257 21583352

[bb5] Nonius (2002). *COLLECT* Nonius BV, Delft, The Netherlands.

[bb6] Otwinowski, Z. & Minor, W. (1997). *Methods in Enzymology*, Vol. 276, *Macromolecular Crystallography*, Part A, edited by C. W. Carter Jr & R. M. Sweet, pp. 307–326. New York: Academic Press.

[bb7] Sheldrick, G. M. (2008). *Acta Cryst.* A**64**, 112–122. 10.1107/S0108767307043930 18156677

[bb8] Spek, A. L. (2009). *Acta Cryst.* D**65**, 148–155. 10.1107/S090744490804362X PMC2631630 19171970

